# Hydric and Thermal Traits of Northern Australian Geckos: Water Loss Is Not Explained by Aridity

**DOI:** 10.1002/ece3.71585

**Published:** 2025-06-17

**Authors:** Kade Skelton, Craig Moritz, Kimberley A. Day, Chava L. Weitzman, Stephen M. Zozaya, Christine Schlesinger, Michael Kearney, Keith A. Christian

**Affiliations:** ^1^ Research Institute for the Environment and Livelihoods Charles Darwin University Darwin Northern Territory Australia; ^2^ Division of Ecology and Evolution, Research School of Biology, and Centre for Biodiversity Analysis The Australian National University Acton Australian Capital Territory Australia; ^3^ School of Biosciences The University of Melbourne Parkville Victoria Australia

**Keywords:** aridity, climate, evaporative water loss, *Gehyra*, phylogeny, thermal preference

## Abstract

Behavioural and physiological adaptations are important for maintaining stable hydric states and viable body temperatures under challenging conditions experienced in variable terrestrial environments. For example, reptiles from arid locations tend to have lower rates of evaporative water loss (EWL). Here we test the prediction that geckos adapt their physiology to match the local environment to reduce hydric and thermal stress. Specifically, we compared EWL and preferred temperature among closely related species living under a range of climatic conditions. EWL rates were measured using a flow‐through system in 18 species in the genus *Gehyra* collected from 11 locations across a strong gradient in aridity in tropical northern Australia during the dry season (Austral winter), and preferred temperatures were measured for nine of these species. Rates of EWL did not differ significantly among most species except between those with the highest and lowest rates. There was no association between EWL and the aridity of the locations where geckos were captured, and microhabitat conditions (temperature and humidity in rock crevices, used as retreats) did not explain this lack of association. Thermal preferences differed among species, with *G. koira* selecting significantly cooler temperatures than all other species. *Gehyra moritzi*, from the most arid and hottest location (Kurundi Station), had the highest preferred body temperature, overlapping only with two sympatric species ( *G. minuta* and *G. purpurascens* ). Unlike some reptiles, there was no evidence *Gehyra* geckos specialise in their EWL to match the local climate despite the strong gradient in aridity across our sampling sites. Nocturnal activity or seasonal plasticity in EWL may explain the lack of association between physiological traits of these species and the broad climatic conditions in the places they live.

## Graphical abstract

Unlike some reptiles, there was no evidence that Gehyra geckos specialise in their EWL to match the local climate despite the strong gradient in aridity across our sampling sites. Nocturnal activity or seasonal plasticity in EWL may explain the lack of association between the physiological traits of these species and the broad climatic conditions in the places they live.

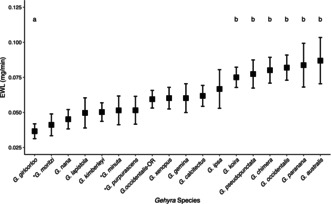

## 1 Introduction

Terrestrial reptiles are challenged with maintaining stable thermal and hydric states in the variable environments they inhabit. Although they rely on environmental temperatures to regulate body temperatures and metabolic processes, with warmer temperatures generally enabling increased sprint speed and digestion efficiency, exposure to extreme thermal conditions can be lethal (Christian and Tracy 1981; Harwood 1979; Hertz et al. 1982). When water loss exceeds water gain, the ensuing hydric stress results in dehydration, with both sublethal and lethal consequences (Pirtle et al. 2019).

Reptiles can overcome thermal and hydric stress by adapting to local conditions. For example, reptiles from warmer environments tend to select higher body temperatures, reflecting physiological and behavioural adaptations to local conditions (Clusella‐Trullas and Chown 2014). Measuring selected body temperatures in a controlled setting is useful for determining the preferred body temperatures of reptiles when they are unaffected by environmental variables that may restrict thermoregulatory behaviours in the field (Hertz et al. 1993). Comparing this measure across species from varying environments can then indicate the extent to which species have adapted to their local conditions.

Reptiles are, to some extent, preadapted to arid environments (Bradshaw 1986), and species that live in arid climates typically have lower evaporative water loss (EWL) rates than those from more mesic regions (Belasen et al. 2017; Bentley and Schmidt‐Nielsen 1966; Cox and Cox 2015; Dmi'el 1998, 2001; Dmi'el et al. 1997; Le Galliard et al. 2021; Mautz 1982a; Shoemaker and Nagy 1977). Cutaneous EWL accounts for most of the non‐excretory water loss in reptiles, and the rate of EWL is dependent on various factors including environmental humidity and skin permeability (Bentley and Schmidt‐Nielsen 1966; Mautz 1982a; Roberts and Lillywhite 1983; Shoemaker and Nagy 1977; Snyder 1979), the latter of which is subject to selective pressures. The differences in EWL of species across environmental gradients suggest that species that persist in arid environments where they are subject to dry conditions and high levels of hydric stress over many generations have evolved to reduce the amount of water passively lost across all surface membranes (Cox and Cox 2015). Historically, colonization of arid environments by reptiles is associated with adaptive changes in EWL (skin permeability), indicating that this process is driven by climate even where a phylogenetic signal is present (Cox and Cox 2015). However, studies of Australian geckos have found no such association (sampling that included one species shared with the present study), possibly because their nocturnal habit protects them from daytime aridity extremes (Vucko 2008; Withers et al. 2000). Also, it is possible that seasonal changes in EWL may represent an alternative physiological adaptation that precludes the advantages of a fixed association between EWL and aridity (Blamires and Christian 1999; Day et al. 2025).

Identifying whether species have adapted to their environment through specialisation of their physiological traits can provide information on species' evolutionary history of dispersal and trait development, explain the co‐existence of sympatric species and, when combined with phylogenetic data, predict how species will cope with future environmental changes (Garcia‐Porta et al. 2019; Piantoni et al. 2019; Sannolo et al. 2018). This approach is especially effective if multiple congeneric species from varied climatic habitats are measured, allowing for consideration of both phylogenetic and ecological influences on physiological traits.

The evolutionary history of the Australian *Gehyra* species complex (dtellas—a First Nations name for this group (Broome 1897)) has recently been re‐evaluated and new species identified (Hutchinson et al. 2014; Doughty et al. 2018; Oliver et al. 2020). This genus is found across mainland Australia, under a range of environmental conditions, particularly with respect to aridity and environmental temperatures, making the group valuable for testing predictions about the extent of physiological specialisation.

**FIGURE 1:**
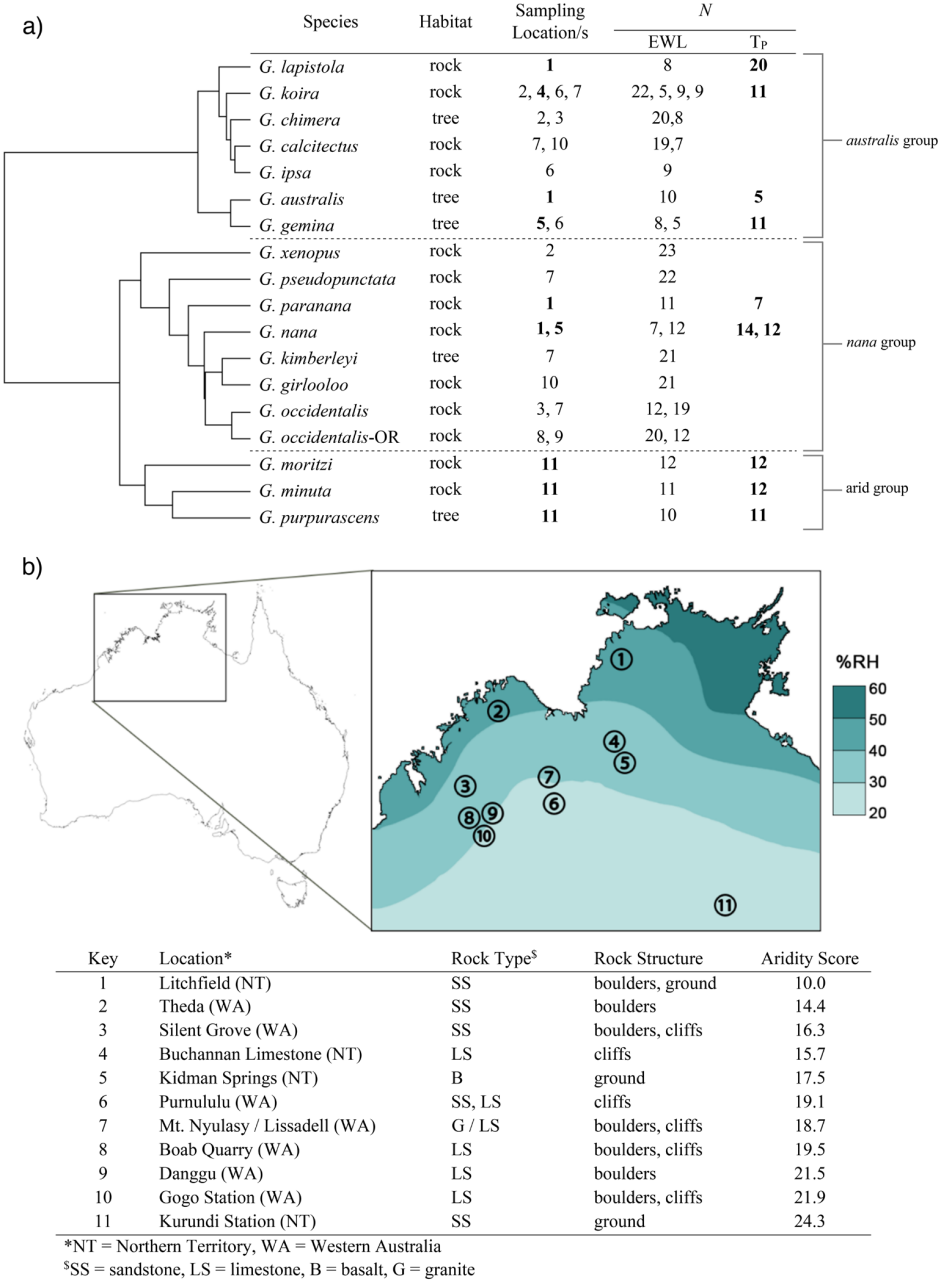
(a) Phylogenetic tree (Lau et al. 2025) and corresponding sampling locations for *Gehyra* geckos sampled during the dry season between 2019 and 2021 for evaporative water loss (EWL) and thermal preference (T_Pref_) analyses. Sampling locations correspond with (b); bold locations in (a) indicate sites for T_Pref_ analyses in addition to EWL analyses. (b) Map of sampling locations and habitat types in the Northern Territory and Western Australia. For context, the map color overlap represents mean annual 3 PM relative humidity (%RH) between 1976 and 2005. Figure modified from map provided by the Australian Bureau of Meteorology, http://www.bom.gov.au/. See Appendix 1; Figure A3 for an alternative map using a color overlap based on vapor pressure (Pa).

We compared physiological traits, measured either in the field or immediately after their relocation to the laboratory, in *Gehyra* from different locations across northern Australia (Western Australia and the Northern Territory; Figure 1). This region is generally characterized by a seasonal tropical climate, with a wet (high humidity and rainfall) and dry (low humidity and rainfall) season. However, conditions range from mesic to arid as latitude increases, such that the more mesic locations experience only minor shifts in environmental temperature diurnally and annually, whereas the more arid locations experience greater changes in temperature at these timescales. At higher latitudes, rainfall and humidity are also lower and the dry season more extreme.

Despite there being no previously established association between EWL and aridity in Australian geckos (Withers et al. 2000; Vucko 2008), the diversity of species and climates included in our study, along with a quantitative metric of aridity, allowed for a stronger test for such an association. We predicted that environmental conditions, even after accounting for phylogeny, would be a driving force behind species differences in EWL and preferred temperatures.

## 2 Materials and Methods

### 2.1 Sample Species and Sites

**FIGURE A1:**
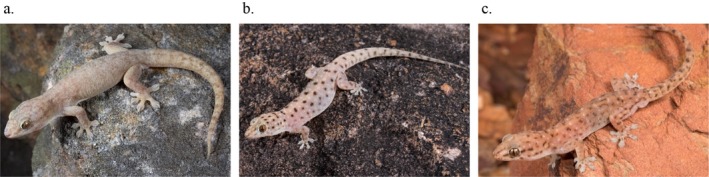
Species of the three *Gehyra* clades represented in this study, including: (a) the *G. australis* clade, characterized by large bodies and preferring trees and rock boulder habitats (photo: *G. lapistola*); (b) the small‐bodied *G. nana* clade, which inhabits various rocky environments (photo: *G. nana* ); and (c) the arid radiation of *Gehyra* (photo: *G. minuta* ). Photos: Stephen Zozaya.

**FIGURE A2:**
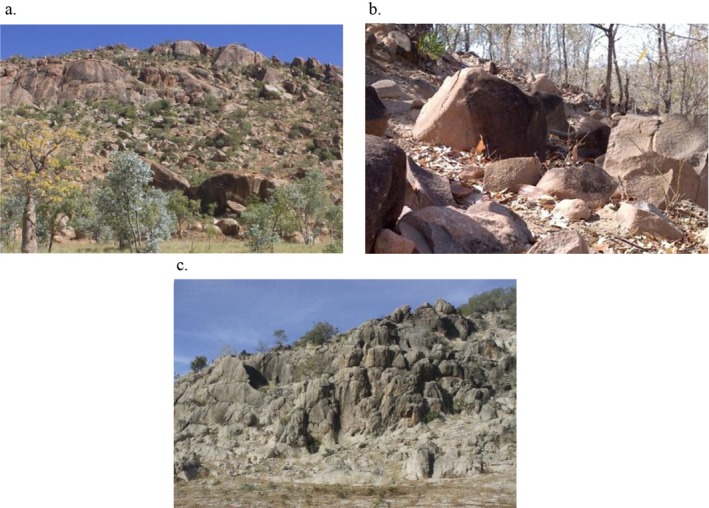
Habitat terrain types of rock‐dwelling north Australian *Gehyra*, including (a) granite boulders, cliffs (Mt. Nyulasy), (b) small, fractured basalt ground rocks (Kidman Springs), and (c) limestone outcrops (Gogo Station).

We sampled 18 *Gehyra* species from 11 locations in Australia's Northern Territory and the northern part of Western Australia between 2019 and 2021 (Figure 1). This region has large‐scale seasonal variation in water availability between the wet and dry seasons, with modest seasonal differences in environmental temperatures (Day et al. 2025; Skelton et al. 2025). To facilitate species comparisons without the complication of seasonal effects, the EWL of all the geckos reported here was measured during the dry season (May to September). Sampled species encompassed the breadth of phylogenetic diversity in Australian *Gehyra*, spanning the three major clades (Figure 1a, Appendix 1; Figure A1): the *G. australis* clade of northern and eastern Australia, the small‐bodied *G. nana* clade of northern Australia, and the “arid” clade, comprised of medium‐to‐small bodied species that occur over a range of habitat types. The species identity of all individuals was confirmed by mtDNA sequencing, which provides reliable diagnoses for the sampled taxa, some of which are difficult to distinguish morphologically (Doughty et al. 2018; Oliver et al. 2020). Although five species are tree‐dwelling, most species are rock‐dwelling, with the type of rock habitat varying across locations (Appendix 1; Figures A1 and A2). Spotlighting methods were used to locate and capture dtellas at night. Spotlighting sessions typically lasted 4 h, from the early evening until sightings diminished. Spotlighting was repeated on consecutive nights with the aim of acquiring at least 10 of each target species from each location. Hatchlings and dtellas with short, incomplete tails or broken skin were excluded from the study and released if captured. All other dtellas were captured by hand and immediately transferred into individual cloth bags. For all species from all sampling sites, field EWL measurements were taken within 48 h of capture; species from Western Australia were measured in the field, species from Kidman Springs were measured in makeshift laboratory conditions, and all other species from the Northern Territory were transported to Charles Darwin University and measured in a laboratory using the same equipment used in the field.

The nine species sampled from the Northern Territory were also used in laboratory‐based thermal preference experiments. Western Australian species were excluded from thermal preference experiments due to logistical challenges related to interstate transport for laboratory‐based experiments. After initial measurements were acquired, dtellas were housed individually in clear plastic holding enclosures of 40 × 25 × 13.5 cm (for moderate/large species) or 17 × 23 × 15 cm (for small species) containing a plastic hide and were supplied with a spray of clean water daily and offered live food three times per week. Dtellas were kept in these enclosures when not used in experiments and were released at their original location after the experiments were completed.

Sampling sites (Figure 1b) were assigned numeric aridity scores calculated through biophysical modelling using NicheMapR (Kearney and Porter 2017, 2020) that integrated humidity and environmental temperatures (see Data S1 for R script). Daily 5 km resolution gridded data on vapour pressure, air temperature, solar radiation, and rainfall from the SILO database (Jeffrey et al. 2001) were used to simulate surface microclimates (Kearney and Porter 2017) at each site from 1999 to 2018 via the micro_silo function of NicheMapR. The SILO dataset does not include wind speed, so the mean monthly wind speed for each location was interpolated to daily from the New et al. (2002) dataset. A substrate of rock was assumed by setting the bulk density equal to the mineral density, and no organic layer was assumed on the surface. Substrate solar absorptivity was assumed to be 0.8, and ‘local’ air temperature, relative humidity, and wind speed were simulated at a height of 0.5 cm. All other settings of micro_silo were left as default values. The microclimate output was then used to run the NicheMapR ectotherm model (Kearney and Porter 2020) to calculate water loss of geckos. A gecko of 5 g, a skin wetness of 0.25%, with no ocular water loss, was simulated to remain outside in the open every nighttime hour. The ectotherm model computes the total water loss from cutaneous and respiratory sources (Kearney and Porter 2020) and these values were summed to obtain annual totals, assuming no water was lost on rainy days or during the daytime hours, and then averaged across years per site to obtain the final aridity score (g water lost/year). Thus, aridity scores reflect both the evaporative potential of the nocturnal environment experienced by active geckos and local rainfall patterns. Higher scores indicate greater aridity.

To obtain direct measurements of microhabitat conditions, iButton temperature/humidity loggers (DS1923 Hygrochrons) were deployed in 2019 at Gogo Station, Kidman Springs, Buchannan Limestone, Litchfield, and Silent Grove sites, which collectively span the aridity gradient. At least four loggers were deployed at each site, with loggers positioned within crevices either known to be occupied by a dtella or similar to known crevices. Also, we positioned one logger at each site outside crevices in shaded locations, including open rock chasms and tree branches. Data retrieval was not successful in all cases, with only a single logger (from a crevice) retrieved at the Silent Grove site due to the impact of fires. Readings were taken hourly; for each logger, we calculated monthly mean, mean maximum, and mean minimum temperature and absolute humidity (calculated from relative humidity and air temperature) readings from inside crevice microhabitats for visualisation and analyses comparing conditions among sites. We also calculated a value representing the range of temperature and relative humidity per month as the difference between the mean maximum and minimum values. Most loggers collected data for 11 or more months. We ensured year‐round data collection from Litchfield by replacing loggers mid‐year, amounting to up to four values per month in open habitat and up to 11 values per month in crevices.

Monthly mean maximum and minimum temperatures from the nearest weather stations (< 100 km from sampling sites) for Northern Territory sampling sites are shown in Table 1 for the wet and dry seasons for the years 2000–2009 inclusive. These values were used in thermal preference comparisons. The Buchannan Limestone location was treated as a Kidman Springs site for this analysis.

**TABLE 1:** Average mean monthly temperatures during dry (May–September) and wet (October–April) seasons in Northern Territory sampling sites between 2000 and 2009 inclusive and the weather stations from which temperature data was acquired (Ali Curung missing 2009 data). Data acquired from the Australian Bureau of Meteorology, http://www.bom.gov.au/climate.

Sampling site	Weather station	Mean monthly minimum temperature (°C)		Mean monthly maximum temperature (°C)	
		Dry season	Wet season	Dry season	Wet season
Litchfield	Batchelor Airport	18.5	23.4	33.9	33.8
Kidman Springs	Kidman Springs Station	15.9	24.0	33.3	36.5
Kurundi Station	Ali Curung	11.3	21.6	28.4	36.0

### 2.2 Evaporative Water Loss

Evaporative water loss (EWL) was measured with an open‐flow system similar to setups used in previous studies (Blamires and Christian 1999; Kosmala et al. 2020; Mautz 1982b; Sadowski‐Fugitt et al. 2012; Tracy et al. 2008; Young et al. 2005). This approach to EWL measurement ensured that factors that may influence water loss rates were controlled (Mautz 1980). Air flow rate and temperature were maintained at constant levels, and measurements were taken from dtellas at rest. The EWL rate includes evaporation across the skin, cloaca, from the eyes, and from respiratory water loss, and these components were not delineated (Mautz 1982b). A size‐independent measure of total water loss among reptiles of different sizes can be approximated by expressing the total EWL as a function of surface area (SA, in cm^2^) of the whole animal (Mautz 1982a). Surface area was estimated for each individual based on its mass with the TrenchR package (Buckley et al. 2023).

In the experimental setup, air was drawn through a dehydration column of silica gel and into a cylindrical experimental chamber (12.5 cm long, 3 cm diameter, 70 mL volume) at a rate of 0.2 L/min using a low‐flow air pump (Sensidyne Gilian LFS‐113D). This chamber was contained within an A&E Lab 18 L portable incubator (model AE‐PI‐100) set to 30°C to maintain a nominal experimental temperature of 30°C (the exact air temperatures were measured by the probes). A Vaisala HUMICAP Humidity and Temperature Probe HMP110 (0–100 ± 1.5% RH, −40 to 80°C ± 0.1°C) downstream of the animal chamber was connected to an ADInstruments PowerLab data acquisition system that continuously recorded the temperature and relative humidity of the air exiting the chamber. Baseline measurements were taken from stable readings before an animal was introduced into the chamber.

After a stable baseline was recorded, an individual dtella was placed in the experimental chamber. The air temperature and relative humidity of the air output was monitored until readings stabilised and the animal remained at rest for 10 min, as evidenced by a stable humidity trace. The humidity sensors were sensitive enough to detect even slight movements. The lowest humidity reading was taken during this rest period, provided humidity remained similarly low for at least 2 min. The difference between the humidity reading with a dtella in the chamber and the baseline reading is a measure of the amount of water lost via evaporation. EWL was calculated from the equations of Bernstein et al. (1977) for an open‐flow system, in conjunction with calculations (List 1971) of saturation vapour density (needed to calculate the mass of water from the measurements of relative humidity). Trials were restricted to 2 h maximum duration to ensure the welfare of the animals used in the experiment, but in most cases the animals had settled and resting values were reached in approximately 1.5 h. After experiments, a dial calliper was used to measure the length (snout‐to‐vent) and width of the body, limbs and tail of dtellas to 0.1 mm, and body mass was weighed to 0.01 g.

### 2.3 Thermal Preference

**FIGURE A4:**
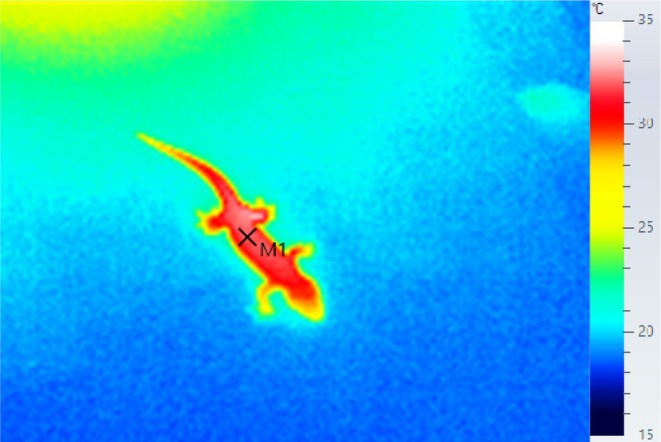
Thermal image of a *Gehyra* showing the temperature sampling site in the lower abdominal region (M1) and the corresponding temperature scale as displayed in the Testo IRSoft thermal imaging software (v4.8) interface. Image taken using a Testo 868 thermal imaging camera (0.08°C thermal sensitivity).

Thermal preference (T_Pref_) experiments were conducted soon after field EWL was measured, within 72 h of collection of animals from the field. An individual dtella was placed in a 60 × 30 × 35 cm glass tank with an artificial crevice made from a 55 × 15 cm length of ceramic tile elevated 1.5 cm by small terracotta blocks. The tank was kept in a temperature‐controlled room set to 20°C, and a 50 W heat globe was placed at one end of the tank to create a linear temperature gradient of 20°C–40°C in a design similar to those used in other studies (Belasen et al. 2017; Christian et al. 1998, 2007; Rozen‐Rechels et al. 2020). A Testo 868 thermal imaging camera (0.08°C thermal sensitivity) was used to photograph the dtella at hourly intervals, producing a total of 12 thermal images for each dtella. Testo IRSoft thermal imaging software (v4.8) was used to extract temperature readings from the lower abdomen of the animal (Appendix 1; Figure A4). This location was selected to replicate the standard procedure of measuring reptile body temperatures via the cloaca.

The central 50% of readings were used to produce a set‐point range which considers the variation in selection of temperatures between upper and lower set‐points; this is standard practice in studies of thermal preference and allows for a more complete capture of thermoregulatory decisions (Hertz et al. 1993; Pintor et al. 2016; Stellatelli et al. 2018).

Initial results indicated that *G. koira* had the lowest T_Pref_ of the nine species measured. Given that this was the only species of the nine that was collected from limestone, this result raised the possibility that the deeper crevices and fissures we observed in the limestone may allow lower daytime body temperatures, resulting in a low T_Pref_. To test this hypothesis, in November 2023 we collected a sample of 12 *G. koira* from each of two sites: the Buchannan Limestone and a site 65 km to the NNW near Timber Creek, NT where *G. koira* were found on sandstone. These two samples of *G. koira* were taken to the laboratory; T_Pref_ was measured as described above, and the two groups were compared as described below.

### 2.4 Statistical Analysis

Analyses were performed with R v4.4.2 in RStudio v2024.09.0 (R Core Team 2024; R Studio Team 2024). We performed type II tests using *Anova* (car package, Fox and Weisberg 2019) to calculate *F* statistics and *p*‐values for non‐phylogenetic linear models, and Wald's *χ* ^2^ tests used for mixed‐effects models. Where relevant, pairwise contrasts were done with *emmeans* (Lenth 2024) using the Tukey method for *p*‐value adjustments.

With the iButton data from five sampling locations, we used linear mixed‐effects models with the *lmer* function in the lme4 package (Bates et al. 2015) to test whether crevice temperature and humidity differ among sites. We analyzed each of the temperature and humidity metrics separately, including month as a covariate and logger ID as a random factor. We had enough iButtons deployed at Litchfield to also compare open and crevice microhabitats for each of the climate metrics with *lmer*.

Data were used to test whether EWL rates differed: (i) within a species at different sites, (ii) among species, and (iii) whether there is a relationship among species between EWL and the aridity or annual rainfall (as determined from the SILO database; Jeffrey et al. 2001) of the capture location. To normalize the distribution of EWL, the values were log‐transformed prior to analyses, and all analyses of EWL include SA as a covariate, removing issues introduced by analyzing a ratio such as EWL/SA (Packard and Boardman 1988). For the seven species sampled at multiple sites (Figure 1), we tested whether EWL values differed among sites using a linear model for each species. Because site was never significant, we focused further analysis on species‐level differences. We tested whether EWL differed among species using a linear model and performed pairwise contrasts of SA‐adjusted EWL among all species pairs. Surface area‐adjusted mean EWL values (using emmeans) were used for visualization.

We used phylogenetic mixed‐effects models to test whether among‐species variation in EWL was associated with aridity or annual rainfall, accounting for phylogenetic non‐independence. These analyses were performed using the R package MCMCglmm (Hadfield 2010). We included EWL as the response variable, SA as a covariate, and either aridity or annual rainfall as the predictor variables of interest. Individuals were nested within species as one random effect, with phylogenetic covariance among species included as another random effect. Phylogenetic covariance was calculated using the inverseA function with the multispecies coalescent phylogeny from Lau et al. (2025), which we pruned to include only the 18 species sampled here. For each analysis, a Markov Chain Monte Carlo (MCMC) chain was run for 420,000 iterations with a thinning interval of 100 following a burn‐in of 20,000 iterations, which yielded posterior distributions of 4000 samples. Every analysis was run twice and assessed for convergence using the gelman.diag function. Weakly informative inverse‐Wishart priors (*V* = 1, nu = 0.002) were used for fixed effects and parameter expanded priors (F1,1, scale = 1000) were used for random effects. Fixed effects were considered significant if the 95% credible interval did not overlap zero. Because our power to detect phylogenetic signal with relatively few species would be low (Blomberg et al. 2003), we limited analyses to accounting for phylogeny, rather than estimating signal.

Repeated‐measures ANOVAs were used to analyse T_Pref_ with *aov*, including individual gecko ID in the error term. We first verified that the two sampled populations of *G. nana* did not differ, and then tested for differences among species in the main dry season data set. Lastly, we tested for differences between two *G. koira* sites in data collected in November 2023.

## 3 Results

### 3.1 Crevice Conditions

**FIGURE 2:**
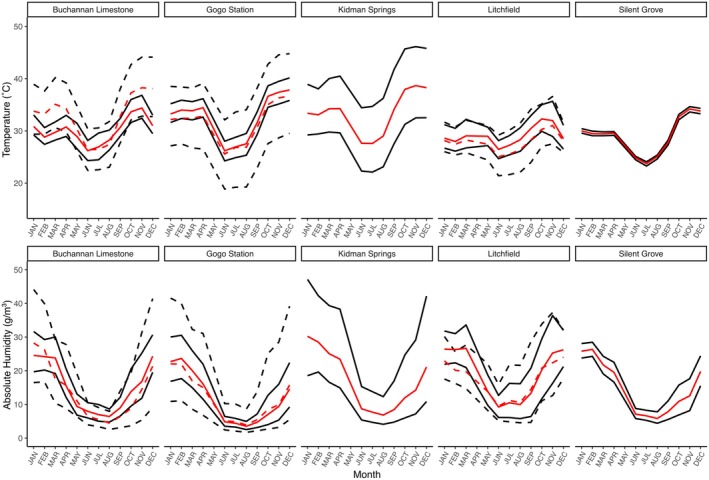
Mean monthly temperature and humidity measured by data loggers placed across sites between 2019 and 2021. Red lines are grand mean values; black lines are mean maximum and mean minimum values. Solid lines represent data collected from inside crevice microhabitats, dashed lines represent data collected from the external environment in the shade. Significant differences in measures between crevice and external conditions were found in Gogo Station (maximum temperature, minimum temperature, maximum humidity, minimum humidity) and Litchfield (mean temperature, minimum temperature).

Mean and mean maximum crevice temperatures measured by the data loggers were significantly different among locations (mean: *χ* ^2^ = 20.0, df = 4, *p* = 0.0005; maximum: *χ* ^2^ = 25.0, df = 4, *p* < 0.0001; Figure 2; Appendix 1; Tables A1 and A2), though mean minimum crevice temperatures did not differ among locations (*χ* ^2^ = 7.2, df = 4, *p* = 0.1). Humidity metrics also differed among the sampling locations (mean: *χ* ^2^ = 99.0, df = 4, *p* < 0.0001; minimum: *χ* ^2^ = 20.1, df = 4, *p* = 0.0005; maximum: *χ* ^2^ = 33.5, df = 4, *p* < 0.0001). Gogo Station crevices maintained lower mean humidity than all other sites except Silent Grove (11.9 g/m^3^ compared to 15.7–17.8 g/m^3^), and Kidman Springs crevices had significantly higher mean maximum temperatures than all sites except Gogo Station (40.2°C compared to 29.5°C–32.2°C).

The monthly ranges of humidity (1.3–40.2 g/m^3^) and temperature (0.5°C–19.5°C) differed across locations (humidity: *χ* ^2^ = 11.6, df = 4, *p* = 0.02; temperature: *χ* ^2^ = 17.1, df = 4, *p* < 0.002). In *post hoc* tests, there were no pairwise differences in the range of humidity between sites. Kidman Springs had a significantly greater temperature range than the other sites (excluding Silent Grove), which may be driven by rock type; the arid limestone sites of Gogo Station and Buchannan Limestone each had a mean range of crevice temperature of ~4°C, whereas the similarly arid Kidman Springs site had a greater mean range of 12°C in crevice temperature and is characterized by small, fractured basalt rocks on the ground.

Generally, crevices provided conditions within the range of temperatures and humidity in open environments (Figure 2). At Litchfield, we deployed enough loggers to compare crevice and open environments and found no significant differences between the two exposure types in all humidity metrics (*χ* ^2^ < 3.25, *p* > 0.07 each). However, most temperature values differed between crevice and open environments (minimum: *χ* ^2^ = 10.8, *p* = 0.001; average: *χ* ^2^ = 10.5, *p* = 0.001; range: *χ* ^2^ = 4.9, *p* = 0.03), indicating that crevices at Litchfield provide higher minimum temperatures, higher average temperatures, and a smaller range of temperatures than open environments. Maximum temperatures between the two environments were similar (*χ* ^2^ = 0.06, *p* = 0.8).

One iButton deployed at Buchannan Limestone was positioned 5 m deep into a crevice, while the four other crevice iButtons were placed < 1 m deep. This deep data logger recorded lower mean monthly minimum, mean, and maximum temperatures than most other crevice loggers from the same site except one with similar mean monthly minimum temperatures. Temperatures in this deep crevice were also lower and more stable than outside conditions at the same site (Table 2).

**TABLE 2:** Monthly mean temperatures recorded by iButton loggers deployed at Buchannan Limestone in varying microhabitat locations between 2019 and 2020. Deep crevice recordings (1 logger) were lower and more stable than shallow crevices (4 loggers) and outside conditions (1 logger).

iButton placement	Minimum temperature (°C)			Mean temperature (°C)			Maximum temperature (°C)		
	Minimum	Maximum	Range	Minimum	Maximum	Range	Minimum	Maximum	Range
Deep crevice	22.4	27.9	5.5	23.4	28.1	4.7	23.8	28.5	4.7
Shallow crevices	24.8	33.6	8.8	26.9	36.0	9.1	29.2	38.9	9.7
Outside shade	22.4	32.8	10.4	26.3	38.3	12.0	30.4	44.1	13.7

### 3.2 Evaporative Water Loss

EWL rates did not differ among sites for any of the seven species that were sampled at multiple localities (*p* ≥ 0.06 in all cases; Table A3). Consequently, we did not include site as a factor in any subsequent models that tested EWL differences among species.

**FIGURE 3:**
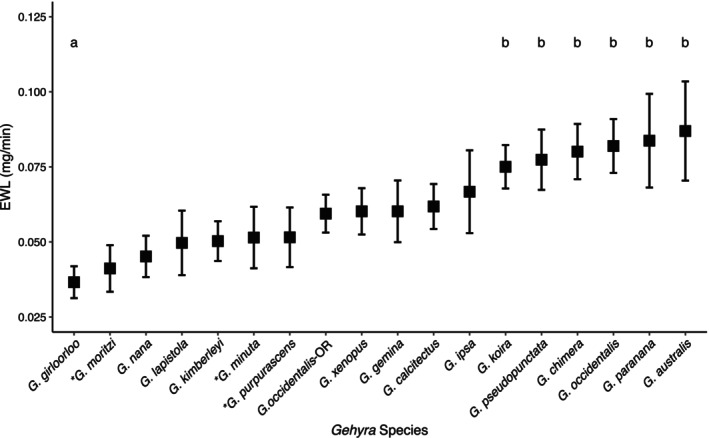
Mean surface area‐adjusted evaporative water loss (EWL) rates of *Gehyra* species sampled in the dry season from the Northern Territory and Western Australia. Data were acquired during May and June, except for species marked with ‘*’ which were sampled in September. Species marked ‘*a*’ are significantly different from species marked ‘*b*’ in *post hoc* emmeans. Error bars display ±1 standard error.

EWL rates differed significantly among species when accounting for SA (F_(17,332)_ = 2.76, *p* = 0.0003). *Post hoc* pairwise contrasts, however, revealed significant differences only between *G. girloorloo*, with the lowest EWL, and the six species at the high end of the EWL range (Figure 3): *G. koira* (*p* = 0.02), *G. pseudopunctata* (*p* = 0.01), *G. chimera* (*p* = 0.007), *G. occidentalis* (*p* = 0.003), *G. paranana* (*p* = 0.03), and *G. australis* (*p* = 0.04). Surface area‐adjusted mean EWL rates ranged from 0.04 to 0.09 mg/min.

**FIGURE A5:**
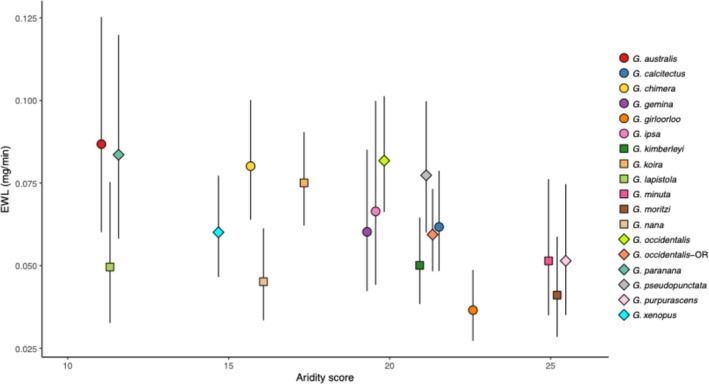
Mean rates of adjusted evaporative water loss (EWL) in relation to the mean aridity score for each of the 18 sampled *Gehyra* species. EWL rates are adjusted to a surface area (SA) of 33.1 cm^2^ (the average SA across the 18 species). Whiskers represent one standard deviation. There was no significant relationship between adjusted EWL and aridity among species.

There was no significant relationship between EWL and aridity score (g water lost/year) when accounting for phylogenetic covariance (Appendix 1; Figure A5; Table A4): with each unit increase in aridity score, EWL was estimated to change by a factor of 0.973 (95% CI = 0.942–1.002). Similarly, there was no significant relationship between EWL and annual rainfall when accounting for phylogenetic covariance (Appendix 1; Table A5): with each 1 mm increase in annual rainfall, EWL was estimated to change by a factor of 1.0002 (95% CI = 0.999–1.0005). The three species from the most arid site (Kurundi, aridity score 24.3) are within the same clade, but there is a large amount of variation among the other *Gehyra* clades. For example, sampled species within the *australis* group are associated with aridity scores ranging from 10.0–21.5 while the *nana* clade ranges from 10.0 to 21.9. Thus, our sampling covers a broad range of aridity values across the two most well‐sampled clades.

### 3.3 Thermal Preference

Preliminary testing determined that there was no significant difference in preferred temperatures between the Litchfield (31.8°C ± 1.06°C) and Kidman Springs (32.5°C ± 1.29°C) populations of *G. nana* (F_(1,24)_ = 2.45, *p* = 0.1). No distinction between these populations is made in the following analysis.

**FIGURE 4:**
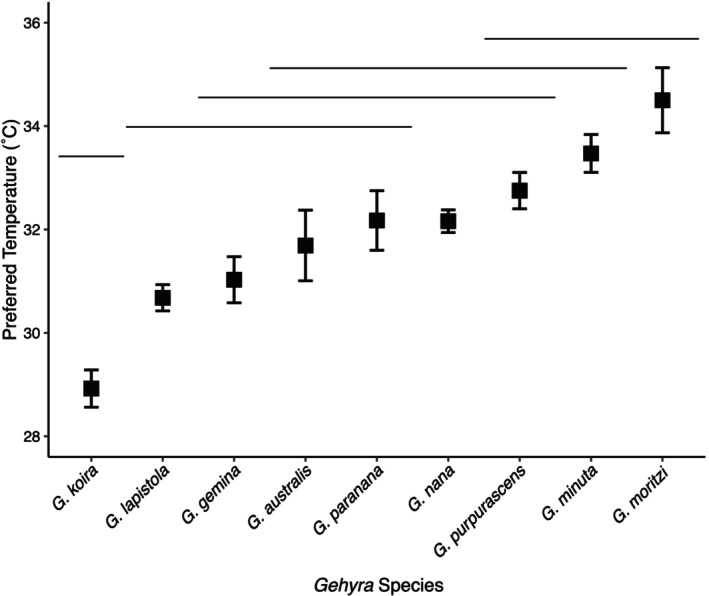
Means of preferred temperatures of *Gehyra* species sampled from locations in the Northern Territory. Measurements were taken during the dry season. Horizontal bars indicate species similarities from *post hoc* tests. Error bars display ±1 standard error.

T_Pref_ was significantly predicted by species (F_(8,106)_ = 17.1, *p* < 0.001; Figure 4). *Post hoc* tests identified that *G. koira* had significantly lower T_Pref_, with a mean preferred temperature of 28.9°C, compared to the means of 30.7°C–34.5°C for the other species. When resampled in 2023, the limestone (32.4°C ± 1.57°C) and sandstone (31.4°C ± 1.15°C) populations of this species were not significantly different with respect to T_Pref_ (F_(1,141)_ = 2.149, *p* = 0.1).

*G. moritzi* had the highest mean T_Pref_ and was significantly different from most species except the other two species from Kurundi Station. Though we did not assess phylogenetic signal in T_Pref_ due to the small number of species measured, it is notable that the three species from this site are all members of the arid group radiation of *Gehyra* (Figure 1a).

## 4 Discussion

### 4.1 Evaporative Water Loss

Evaporative water loss rates differed among *Gehyra* species after accounting for surface area; however, this difference was largely driven by results for a single species, *G. girloorloo*. This result contradicts our prediction that species would differ in their EWL rates and suggests that hydric physiology is not specialised to local habitat conditions in these species.

There was no significant association between EWL and the aridity index or annual rainfall, contradicting our prediction that EWL would be lower in species from more arid locations. If crevice conditions were similar across sites despite differences in broadscale aridity, this could also explain a lack of association between aridity and EWL, as crevices may provide shelter from climatic conditions (Mautz 1982b). But crevice conditions differed between locations and generally did not differ significantly from external conditions, except in the cases of Gogo Station and Litchfield. Thus, neither broad‐scale aridity nor microhabitat conditions greatly affected EWL rates. It is notable when comparing arid sites that the limestone crevices at Gogo Station and Buchannan Limestone provided a more stable climatic environment compared to the shallow microhabitats of the small basalt rocks of Kidman Springs; however, this seems to have little consequence on species' EWL rates.

Thus, although Cox and Cox (2015) concluded that climate is the primary driver of adaptive physiological trait development in arid‐zone reptiles, we did not find a close association between EWL and climate in *Gehyra*. This result aligns with findings in studies of other Australian gecko species, and as suggested in these studies, could be attributed to the nocturnal habit of geckos, which shields them from diurnal extremes in environmental conditions (Vucko 2008; Withers et al. 2000) despite differences among locations with respect to aridity at night. Alternatively, the lack of specialisation in this trait could be explained by physiological plasticity; it is possible that the capacity to shift EWL in response to immediate local, seasonal conditions (Christian et al. 2023; Day et al. 2025) may eliminate the need for specialisation related to habitat aridity.

The three studies that have examined the association between EWL and aridity in Australian geckos (Vucko 2008; Withers et al. 2000; this study) have assessed aridity in different ways, but the lack of an association now includes data from 55 species across a wide taxonomic range of geckos. Future work should address both of the emerging patterns: that the pattern among many reptiles is for decreasing EWL with increasing aridity (references above), and that geckos are an exception to this trend. Studies of diurnal geckos as well as seasonal measurements of geckos from other parts of the world to assess their capacity for acclimatisation could shed light on the generality of these trends and on the reasons why geckos may differ from other reptiles.

### 4.2 Thermal Preference

T_Pref_ differed among species, with the most notable result being *G. koira* 's particularly low T_Pref_. The low T_Pref_ of *G. koira* was significantly different from all other species and was the only mean T_Pref_ less than 30°C. Comparisons between T_Pref_ of *G. koira* sampled in limestone and sandstone locations did not support the hypothesis that the low T_Pref_ of this species was a consequence of inhabiting limestone with deep, cooler crevices.

*G. moritzi* also had a notable T_Pref_, selecting for high temperatures that were similar only to the high T_Pref_ of other species co‐occurring at Kurundi Station. Kurundi Station experiences strong daily fluctuations in temperatures during the dry season, with hot day and cool night temperatures (Table 1). The higher T_Pref_ of species from this site may reflect a tolerance of high daytime temperatures and use of patchy warm microhabitats at night, because small rocks can heat up quickly and retain warmth for relatively long periods of time (Kearney 2002).

For other species, T_Pref_ showed no notable trends. Nocturnal activity patterns may shield dtellas from exposure to diurnal temperature extremes, but they would nevertheless experience daily and seasonal thermal fluctuations. Thermal variability within refugia can allow nocturnal reptiles to thermoregulate (Kearney and Predavec 2000; Nordberg and Schwarzkopf 2019). Thus, dtellas may behaviourally maintain temperatures close to T_Pref_ through selection of microhabitats with favorable temperatures, a pattern of habitat preference that has been observed in other geckos (Shah et al. 2004).

### 4.3 Conclusion

Few differences in EWL were identified across *Gehyra* species during the dry season, suggesting dtellas are not specializing their hydric physiology to their local microhabitats or broadscale climates. This result contrasts with studies in other reptiles that show an inverse relationship between aridity and EWL (Cox and Cox 2015), although our results are consistent with previous studies of other Australian geckos (Vucko 2008; Withers et al. 2000). This pattern may be a result of their nocturnal habits or a result of physiological acclimatization functioning as an alternative to specialization to local conditions. Additional studies of gecko EWL from different climatic regions could help delineate the roles of a nocturnal lifestyle from physiological plasticity in response to large‐scale seasonal variation. Differences in T_Pref_ across species may be driven by local thermal conditions, but there was overlap across species. Crevice conditions dampen extreme temperatures, but at most sites were not otherwise substantially different from external shade conditions. The combination of a nocturnal lifestyle and behavioral thermoregulation within crevices may be sufficient to maintain body temperatures close to a similar T_Pref_ for most species.

## Author Contributions

**Kade Skelton:** conceptualization (supporting), data curation (equal), formal analysis (supporting), investigation (equal), methodology (supporting), writing – original draft (lead), writing – review and editing (equal). **Craig Moritz:** conceptualization (lead), funding acquisition (lead), investigation (equal), project administration (lead), resources (equal), supervision (equal), writing – original draft (supporting), writing – review and editing (equal). **Kimberley A. Day:** data curation (equal), investigation (equal), methodology (equal), project administration (equal), validation (supporting), writing – review and editing (supporting). **Chava L. Weitzman:** data curation (equal), formal analysis (lead), investigation (supporting), supervision (supporting), validation (equal), writing – review and editing (equal). **Stephen M. Zozaya:** data curation (supporting), formal analysis (equal), investigation (equal), methodology (supporting), writing – review and editing (equal). **Christine Schlesinger:** project administration (supporting), supervision (equal), writing – original draft (supporting), writing – review and editing (supporting). **Michael Kearney:** conceptualization (equal), formal analysis (supporting), investigation (supporting), methodology (supporting), software (equal), visualization (supporting), writing – review and editing (supporting). **Keith A. Christian:** conceptualization (lead), funding acquisition (lead), investigation (equal), methodology (equal), project administration (lead), resources (lead), supervision (lead), writing – original draft (supporting), writing – review and editing (equal).

## Conflicts of Interest

The authors declare no conflicts of interest.

## Supporting information


Data S1.

## Figures and Tables

**FIGURE A3:**
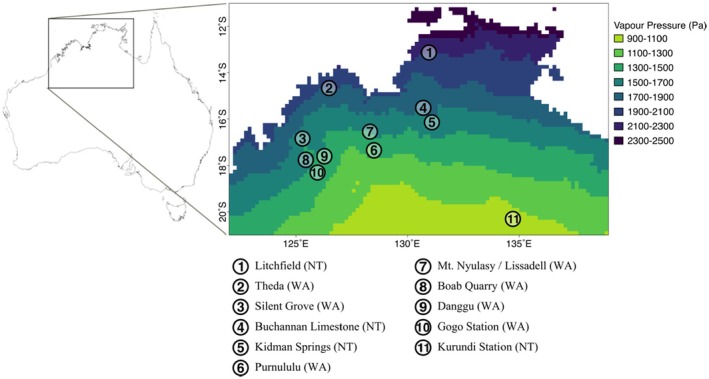
Sites in the Northern Territory (NT) and Western Australia (WA) where *Gehyra* geckos were sampled. The background map is colour‐coded by vapor pressure, calculated with data from New et al. (2002), which provides average values for 1960–1990.

## Data Availability

Data are available from Figshare: https://doi.org/10.6084/m9.figshare.25116158.

## Appendix 1

**TABLE A1:** Mean temperature (°C) and absolute humidity (g/m^3^) readings from environmental data loggers deployed in crevices and shaded open‐air locations across Western Australia and Northern Territory sites.

Location	Measure (means)		Mean value	
			Crevice	Open
Buchannan Limestone	Temperature	Minimum	28.2	28.4
		Mean	30.0	33.0
		Maximum	32.2	38.0
		Range	4.0	9.6
	Absolute humidity	Minimum	12.1	7.6
		Mean	15.7	14.1
		Maximum	20.7	25.0
		Range	8.6	17.4
Gogo Station	Temperature	Minimum	30.7	24.9
		Mean	32.7	31.6
		Maximum	34.6	38.6
		Range	3.9	13.7
	Absolute humidity	Minimum	8.4	5.2
		Mean	11.9	11.8
		Maximum	16.7	25.5
		Range	8.4	20.3
Kidman Springs^a^	Temperature	Minimum	28.1	28.4
		Mean	33.5	33.0
		Maximum	40.2	38.0
		Range	12.1	9.6
	Absolute humidity	Minimum	10.2	7.6
		Mean	16.9	14.1
		Maximum	29.2	25.0
		Range	19.0	17.4
Litchfield	Temperature	Minimum	27.1	25.6
		Mean	29.2	28.4
		Maximum	31.6	32.7
		Range	4.5	7.1
	Absolute humidity	Minimum	12.2	12.8
		Mean	17.8	19.6
		Maximum	24.4	29.3
		Range	12.1	16.5

^a^ Open‐air values for Kidman Springs were sourced from Buchannan Limestone loggers.

**TABLE A2:** Post hoc comparisons of mean temperature (°C) and absolute humidity (g/m^3^) readings of crevices across Western Australia and Northern Territory locations.

Mean values	Silent grove		Litchfield		Kidman springs		Buchannan limestone		Gogo station	
	Temp	Humidity	Temp	Humidity	Temp	Humidity	Temp	Humidity	Temp	Humidity
Gogo Station				*					**30.7**	**8.4**
			*	*		*		*	**32.7**	**11.9**
				*		*			**34.6**	**16.7**
					*				**3.9**	**8.4**
Buchannan Limestone							**28.2**	**12.1**		
				*			**30.0**	**15.7**		
					*	*	**32.2**	**20.7**		
					*		**4.0**	**8.6**		
Kidman Springs					**28.1**	**10.2**				
			*	*	**33.5**	**16.9**				
	*		*		**40.2**	**29.2**				
			*		**12.1**	**19.0**				
Litchfield			**27.1**	**12.2**						
		*	**29.2**	**17.8**						
			**31.6**	**24.4**						
			**4.5**	**12.1**						
Silent Grove	**28.7**	**12.3**								
	**29.1**	**14.9**								
	**29.5**	**17.9**								
	**0.9**	**5.6**								

*Marks a significant difference (*p* < 0.05) between locations. In order per site: Blue = mean minimums, black = grand means, red = mean maximums, and green = range.

**TABLE A3:** Evaporative water loss rates did not differ among sites for the seven species sampled at multiple localities. Results from linear models per species with surface area as a covariate.

Species	Site	Estimated marginal means ± SE	Statistic	*p*
*G. calcitectus*	Gogo Station	0.103 ± 0.0243	F_(1,25)_ = 2.69	0.1
	Mt. Nyulasy/Lissadell	0.065 ± 0.0092		
*G. chimera*	Silent Grove	0.082 ± 0.0097	F_(1,25)_ = 0.86	0.4
	Theda	0.093 ± 0.0069		
*G. gemina*	Kidman Springs	0.070 ± 0.0083	F_(1,10)_ = 0.99	0.3
	Purnululu	0.087 ± 0.0138		
*G. koira*	Buchannan	0.074 ± 0.0149	F_(3,39)_ = 2.69	0.06
	Mt. Nyulasy/Lissadell	0.131 ± 0.0184		
	Purnululu	0.088 ± 0.0131		
	Theda	0.084 ± 0.0075		
*G. nana*	Kidman Springs	0.034 ± 0.0086	F_(1,16)_ = 0.44	0.5
	Litchfield	0.025 ± 0.0087		
*G. occidentalis*	Mt. Nyulasy/Lissadell	0.095 ± 0.0171	F_(1,28)_ = 0.04	0.8
	Silent Grove	0.088 ± 0.0214		
*G. occidentalis* —OR	Boab Quarry	0.057 ± 0.0081	F_(1,29)_ = 0.21	0.7
	Danggu	0.064 ± 0.0117		

**TABLE A4:** Results of the phylogenetic mixed‐effects model testing the relationship between evaporative water loss (EWL) and aridity using *MCMCglmm*. Because EWL was log‐transformed prior to analyses, all posterior estimates are on a log scale.

Parameter	Estimate (*β*)	Lower 95% CI	Upper 95% CI	pMCMC
Intercept	**−3.20812**	**−3.81652**	**−2.49354**	**< 0.0003**
Surface area	**0.02678**	**0.01969**	**0.03352**	**< 0.0003**
Aridity	−0.02737	−0.05983	0.00185	0.0715

*Note:* Statistically significant parameters with 95% credible intervals (CI) that do not intersect zero appear in bold.

**TABLE A5:** Results of the phylogenetic mixed‐effects model testing the relationship between evaporative water loss (EWL) and annual rainfall using *MCMCglmm*. Because EWL was log‐transformed prior to analyses, all posterior estimates are on a log scale.

Parameter	Estimate (*β*)	Lower 95% CI	Upper 95% CI	pMCMC
Intercept	**−3.942**	**−4.437**	**−3.453**	**< 0.0003**
Surface area	**0.027**	**0.0199**	**0.03411**	**< 0.0003**
Rainfall	0.000247	−0.000037	0.000572	0.107
