# Supplementary material for: Hydric and Thermal Traits of Northern Australian Geckos: Water Loss Is Not Explained by Aridity
Source: Ecol Evol. 2025 Jun 17;15(6):e71585. doi: 10.1002/ece3.71585 (PMC12171639; doi:10.1002/ece3.71585)
Supplement: Supplementary file 1 — Data S1. [file ECE3-15-e71585-s001.docx]

**Hydric and Thermal Traits of Northern Australian Geckos: Water Loss is not Explained by Aridity**

Supporting Information

The R script used to produce the aridity index using NicheMapR in conjunction with the SILO database. See main text for references and other details.

=============================

library(NicheMapR)

library(tidyverse)

sitenames <- c("Litchfield", "Kidman", "Buchanan", "Bullo", "Kurindie", "MtNyulasy", "Purnululu", "Geikie", "GoGo", "SilentGrove", "Theda", "MtBarnett", "Boab")

lons <- c(130.805, 130.958, 130.639, 129.659, 134.681, 128.278, 128.406, 125.702, 125.765, 125.248, 126.495, 125.928, 125.299)

lats <- c(-13.125, -16.119, -15.932, -15.659, -20.496, -16.748, -17.462, -18.104, -18.327, -17.068, -14.789, -16.717, -17.915)

# microclimate model parameters

REFL <- 0.2 # substrate solar reflectivity, -

BulkDensity <- 2.55 # soil bulk density, Mg/m3 - close to mineral density to simulate rock

ERR <- 1.5 # model error tolerance

Usrhyt <- 0.05 # height of gecko, m

ystart <- 2017

yfinish <- 2018

nyears <- yfinish - ystart + 1

# loop through sites and run simulations

for(i in 1:length(sitenames)){

loc <- c(lons[i], lats[i])

longlat <- loc

micro <- micro_silo(loc = loc,

ERR = ERR,

dstart = paste0('01/01/', ystart),

dfinish = paste0('31/12/', yfinish),

snowmodel = 0,

runmoist = 0,

Usrhyt = Usrhyt,

REFL = REFL,

cap = 0,

soilgrids = 0,

email = "m.kearney@unimelb.edu.au",

BulkDensity = BulkDensity,

BD = rep(BulkDensity, 19))

saveRDS(micro, file = paste0('micro_SILO_', sitenames[i], ystart, '_', yfinish, '.Rda'))

}

# extract rainfall data and aggregate it

annual.rain <- as.data.frame(matrix(nrow = length(sitenames) * nyears, ncol = 3))

for(i in 1:length(sitenames)){

start <- micro$nyears * (i - 1) + 1

finish <- i * micro$nyears

micro <- readRDS(file = paste0('micro_SILO_', sitenames[i], ystart, '_', yfinish, '.Rda'))

rainfall <- micro$RAINFALL

dates2 <- micro$dates2

rain.year <- as.numeric(as.matrix(aggregate(rainfall, by = list(format(dates2, "%Y")), FUN = 'sum')))

annual.rain[start:finish, 2:3] <- rain.year

annual.rain[start:finish, 1] <- sitenames[i]

}

mean.annual.rain <- aggregate(annual.rain, by = list(annual.rain$V1), FUN = mean)[, c(1, 4)]

colnames(mean.annual.rain) <- c('site', 'rain')

colnames(annual.rain) <- c('site', 'year', 'rain')

write.csv(annual.rain, file = paste0('annual.rain_SILO.csv'))

write.csv(mean.annual.rain, file = paste0('mean.annual.rain_SILO.csv'))

# gecko parameters

Ww_g <- 5 # wet mass, g

shape <- 3 # lizard shape, Dipsosaurus dorsalis morphology

pct_wet <- .25 # skin wetness, fraction

pct_eyes <- 0 # no ocular water loss assumed (permanent spectacle)

# activity

nocturn <- 1

diurn <- 0

crepus <- 0

shade_seek <- 0

mindepth <- 3 # node 3, 5 cm

maxdepth <- 10 # node 10, 2m

# behavioural thresholds (make it so they always come out at night)

CT_min <- -100

T_RB_min <- -100

T_B_min <- -100

T_F_min <- -100

T_F_max <- +100

T_pref <- +100

CT_max <- +100

# simulate ectotherm at all sites

for(i in 1:length(sitenames)){

# load current microclimate model output

micro <- readRDS(file = paste0('micro_SILO_', sitenames[i], ystart, '_', yfinish, '.Rda'))

# run ectotherm simulation

ecto <- ectotherm(Ww_g = Ww_g,

shape = shape,

CT_min = CT_min,

T_RB_min = T_RB_min,

T_B_min = T_B_min,

T_F_min = T_F_min,

T_pref = T_pref,

T_F_max = T_F_max,

CT_max = CT_max,

diurn = diurn,

nocturn = nocturn,

crepus = crepus,

shade_seek = shade_seek,

mindepth = mindepth,

maxdepth = maxdepth,

pct_wet = pct_wet,

pct_eyes = pct_eyes)

# save the output

environ <- as.data.frame(ecto$environ)

masbal <- as.data.frame(ecto$masbal)

metout <- as.data.frame(micro$metout)

rainfall <- micro$RAINFALL

dates <- micro$dates

dates2 <- micro$dates2

# make a rainfall presence-absence vector

rain <- rainfall

rain[rainfall >= 1] <- 0

rain[rainfall < 1] <- 1

# cumulatively sum evaporation, resetting when it rains

cumsum <- matrix(data = 0, nrow = length(dates), ncol = 3)

cumsum <- as.data.frame(cumsum)

colnames(cumsum) <- c('cut', 'rain', 'cum')

cumsum$rain <- 1

cumsum[, 1] <- masbal$H2OCut_g

cumsum[seq(1, length(dates), 24), 2] <- rain

a <- cumsum %>% mutate(rl = rep(1:length(rle(rain)$length), times = rle(rain)$length)) %>%

group_by(rl) %>%

mutate(cum = rain*cumsum(cut)) %>%

ungroup() %>%

dplyr::select(-4)

cumsum <- a

# now aggregate to get the max per site

cumevap <- aggregate(cumsum, by = list(format(dates, "%Y")), FUN = 'max')

# evaporation sum per day and then per year

evap <- aggregate(masbal$H2OCut_g, by = list(format(dates, "%d/%m/%Y")), FUN = 'sum')

evap <- evap[order(as.POSIXct(evap$Group.1, format = "%d/%m/%Y")), ]

evap.yearly <- aggregate(evap$x, by = list(format(dates2, "%Y")), FUN = 'sum')

# recalculate, setting any days where it rained to zero evap

evap2 <- evap

evap2$x[rainfall >= 1] <- 0

evap2.yearly <- aggregate(evap2$x, by = list(format(dates2, "%Y")), FUN = 'sum')

write.csv(evap.yearly, paste0('alwaysout.noct.evap.yearly.', sitenames[i], '_SILO.csv'))

write.csv(evap2.yearly, paste0('alwaysout.noct.evap2.yearly.', sitenames[i], '_SILO.csv'))

write.csv(cumevap, paste0('alwaysout.noct.cumevap.', sitenames[i], '_SILO.csv'))

}

# summarise indices for each site for each year

all.aridity <- as.data.frame(matrix(nrow = length(sitenames) * micro$nyears, ncol = 5))

all.aridity[, 2] <- rep(seq(ystart, yfinish), length(sitenames))

for(i in 1:length(sitenames)){

start <- micro$nyears * (i - 1) + 1

finish <- i * micro$nyears

all.aridity[start:finish, 1] <- sitenames[i]

data.in <- read.csv(paste0('alwaysout.noct.cumevap.', sitenames[i], '_SILO.csv'))

all.aridity[start:finish, 3] <- data.in$cum

data.in <- read.csv(paste0('alwaysout.noct.evap.yearly.', sitenames[i], '_SILO.csv'))

all.aridity[start:finish, 4] <- data.in$x

data.in <- read.csv(paste0('alwaysout.noct.evap2.yearly.', sitenames[i], '_SILO.csv'))

all.aridity[start:finish, 5] <- data.in$x

}

# summarise across years

colnames(all.aridity) <- c('site', 'year', 'all.night.cumulative', 'all.night.totevap', 'all.night.raincorrected')

agg.aridity <- aggregate(all.aridity, by = list(all.aridity$site), FUN = mean)

agg.aridity$site <- agg.aridity$Group.1

agg.aridity <- agg.aridity[, c(-1, -3)]

mean.annual.rain <- read.csv(file = paste0('mean.annual.rain_SILO.csv'))[, -1]

agg.aridity <- merge(agg.aridity, mean.annual.rain)

par(mfrow = c(1, 1))

plot(agg.aridity$all.night.raincorrected ~ agg.aridity$rain, pch = 16, xlab = 'mean annual rainfall, mm', ylab = 'mean total annual evaporation, g')

agg.aridity <- agg.aridity[order(agg.aridity$rain), ]

par(mfrow = c(3, 1))

for(i in 1:3){

barplot(agg.aridity[, i + 1], names = agg.aridity$site, cex.names = .85, las = 2, main = colnames(all.aridity[i + 2]), ylim = c(0, max(agg.aridity[, i + 1] * 1.1)))

box()

}

for(i in 1:4){

if(i %in% 3){

barplot(agg.aridity[, i + 1], names = agg.aridity$site, cex.names = .85, las = 2, main = colnames(all.aridity[i + 2]), ylim = c(0, 210))

}else{

barplot(agg.aridity[, i + 1], names = agg.aridity$site, cex.names = .85, las = 2, main = colnames(all.aridity[i + 2]), ylim = c(0, 50))

}

box()

}

write.csv(agg.aridity, paste0('aridity.summary_SILO.csv'))
